# Activation of neutral sphingomyelinase 2 by starvation induces cell-protective autophagy via an increase in Golgi-localized ceramide

**DOI:** 10.1038/s41419-018-0709-4

**Published:** 2018-06-04

**Authors:** Moon Jung Back, Hae Chan Ha, Zhicheng Fu, Jong Min Choi, Yongwei Piao, Jong Hoon Won, Ji Min Jang, In Chul Shin, Dae Kyong Kim

**Affiliations:** 1 Department of Environmental and Health Chemistry, College of Pharmacy, Chung-Ang University, 84 Heukseok-ro, Dongjak-gu, Seoul 06974 Republic of Korea; 2 Present Address: Department of Molecular and Cellular Biology, Baylor College of Medicine, Houston, TX 77030 USA; 3 Present Address: Department of Biochemistry and Molecular Genetics, College of Medicine, University of Illinois at Chicago, Springfield, IL USA

## Abstract

Autophagy is essential for optimal cell function and survival, and the entire process accompanies membrane dynamics. Ceramides are produced by different enzymes at different cellular membrane sites and mediate differential signaling. However, it remains unclear which ceramide-producing pathways/enzymes participate in autophagy regulation under physiological conditions such as nutrient starvation, and what the underlying mechanisms are. In this study, we demonstrate that among ceramide-producing enzymes, neutral sphingomyelinase 2 (nSMase2) plays a key role in autophagy during nutrient starvation. nSMase2 was rapidly and stably activated upon starvation, and the enzymatic reaction in the Golgi apparatus facilitated autophagy through the activation of p38 MAPK and inhibition of mTOR. Moreover, nSMase2 played a protective role against cellular damage depending on autophagy. These findings suggest that nSMase2 is a novel regulator of autophagy and provide evidence that Golgi-localized ceramides participate in cytoprotective autophagy against starvation.

## Introduction

Autophagy is a highly conserved intracellular catabolic process that degrades cytoplasmic components within lysosomes^1^. Autophagy protects cells under nutrient-deprived conditions by recycling cytoplasmic materials, and removes excess or toxic cellular components^2^. Dysfunction of the homeostatic role of autophagy contributes to the progression of various diseases, including neurodegenerative diseases^3^. The accumulation of toxic protein aggregates is considered the main cause of Parkinson’s disease (PD) and Alzheimer’s disease (AD)^4^. Thus, autophagy-regulating molecules are attractive therapeutic targets for neurodegenerative diseases.

Autophagy processes accompany membrane dynamics, starting with the initial step of an isolated cup-shaped, double-membrane structure termed the phagophore in the cytoplasm. This phagophore expands in size and eventually engulfs cytoplasmic cargo to form an autophagosome^1^. The autophagosome delivers the cargo to the lysosome by fusing to form an autolysosome, after which the cargo is degraded. During autophagy, the membrane undergoes continuous remodeling including curvature formation, budding, fission, and fusion^5^. The entire autophagy process relies on the regulation of membrane dynamics.

Ceramides are the central molecules of sphingolipid metabolism. They are constituents of cellular organelle membranes that mediate membrane dynamics by altering membrane fluidity or rigidity and also function as signaling molecules^6^. Ceramides are generated via various enzyme-mediated pathways in different cellular organelles. Ceramides can be synthesized by the condensation of serine and palmitoyl-CoA, catalyzed by serine palmitoyl transferase (SPT) and ceramide synthase (CerS; de novo pathway) in the endoplasmic reticulum (ER)^7^. Another ceramide-producing pathway is the sphingomyelin (SM) pathway, in which SM is hydrolyzed to ceramides by neutral sphingomyelinase 2 (nSMase2) in the Golgi complex and plasma membrane^8,9^ or by acidic sphingomyelinase (aSMase) in the lysosome^10^. The ceramide production pathway determines the diverse physiological roles of ceramides in cellular processes, including cell proliferation, differentiation, and apoptosis^11–15^. Ceramides have been proposed to act as regulators of autophagy^16–18^. However, which ceramide-producing pathway participates in autophagy under physiological conditions, such as starvation, remain unclear.

nSMase2 is a Mg^2+^- and phosphatidylserine-dependent neutral form of SMase. It is the major isoform of nSMase^19^ and is highly expressed in the brain and bone^20^. nSMase2 has been studied concerning cell growth and development^20–22^, and plays pivotal role in exosome release via the control of exosome budding into multivesicular endosomes^23,24^. However, the role of nSMase2 in autophagy remains unknown.

In this study, we identified nSMase2 as a key enzyme that mediates starvation-induced autophagic flux, among other ceramide-producing enzymes, through its stable activation and increase in Golgi-localized ceramides. nSMase2 protected cells against various toxic stimuli, including starvation and dopaminergic toxicity. Additionally, the expression of nSMase2 in the brain of old mice with reduced autophagy was decreased compared with that in young mice. In GEO database analysis, the expression of nSMase2 in the substantia nigra was significantly lower in patients with PD than in healthy donors, and was correlated with autophagy-related genes (ATGs). Overall, these findings demonstrate that nSMase2 mediates autophagy and has a cytoprotective role, providing essential insights into the complexity of the mechanisms underlying autophagy regulation. This further suggests that nSMase2 may serve as a novel therapeutic target for treating autophagy-associated diseases, especially PD.

## Results

### nSMase2 mediates starvation-induced autophagy

**Fig. 1:**
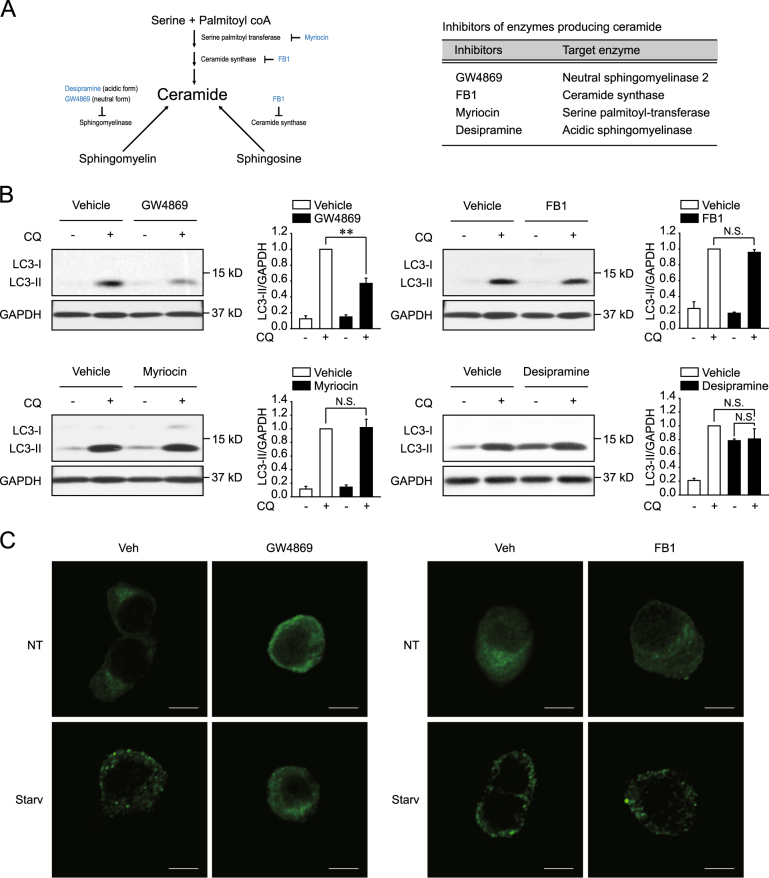
GW4869 suppresses starvation-induced autophagy. **a** Inhibitors of ceramide-producing enzymes. **b** Modulation of LC3 turnover by treatment with the nSMase2 inhibitor GW4869. PC12 cells were untreated or pretreated with each agent (5 μM GW4869, 10 μM FB1, 5 μM myriocin, 10 μM desipramine, or vehicle) for 1 h and then starved with each inhibitor in the presence or absence of 50 μM chloroquine (CQ) for 2 h. LC3 and glyceraldehyde-3-phosphate dehydrogenase (GAPDH) were analyzed by immunoblot assay. LC3-II levels were normalized to GAPDH levels. The data are presented as the mean ± SEM of three independent experiments. Significant differences, **p < 0.01; NS, not significant **c** Reduced LC3 puncta formation by GW4869. PC12 cells were pretreated with each agent (5 μM GW4869, 10 μM FB1, or the vehicle) for 1 h and then incubated with HBSS or growth medium for 2 h with each inhibitor or vehicle. LC3 puncta were detected by immunofluorescence assay using anti-LC3 antibody. Scale bar = 5 μm

To investigate the ceramide-producing enzyme or pathway involved in the regulation of autophagy, autophagic flux was analyzed in the presence of various inhibitors of ceramide-generating enzymes (Fig. 1a) in rat neuroblastoma PC12 cells, which feature a well-conserved sphingolipid metabolism (Supplementary Figure 1A). Autophagic flux was induced by starvation in Hank’s balanced salt solution (HBSS) and was measured by determining the turnover of LC3. The assay compares the levels of the autophagosome marker, LC3-II, in the presence or absence of the lysosomal inhibitor, chloroquine (CQ), to estimate the degree of autophagic LC3 degradation^25^. Starvation-induced LC3 turnover was suppressed by the nSMase2 inhibitor GW4869 (Fig. 1b). Furthermore, starvation-induced LC3 puncta formation, which is associated with autophagosome or autolysosome formation, was diminished by treatment with GW4869 (Fig. 1c). However, the de novo pathway inhibitors, CerS inhibitor (fumonisin B1) and SPT inhibitor (myriocin), had negligible effect on starvation-induced LC3 turnover (Fig. 1b). Treatment with the aSMase inhibitor desipramine increased LC3-II levels in the absence of CQ, while LC3-II levels in the presence of CQ were similar to those of the vehicle-treated control group (Fig. 1b). The data indicate that the inhibitor acted as a lysosomal inhibitor. Thus, aSMase may be required for lysosomal function rather than for autophagic flux, which is consistent with results from previous studies ^26,27^.

**Fig. 2:**
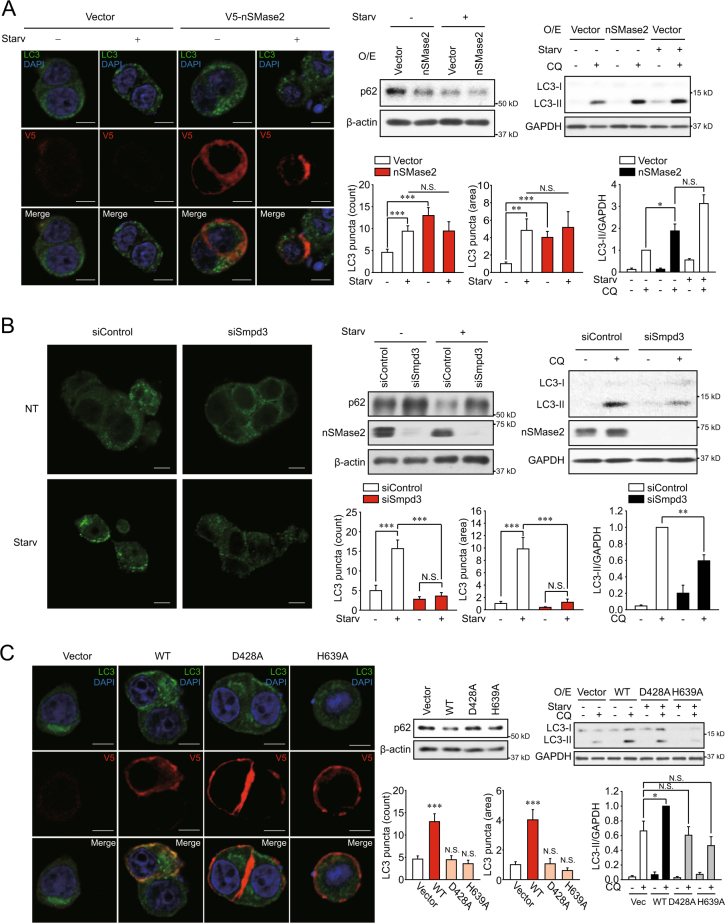
nSMase2 induces autophagy. **a** Enhanced autophagic flux by overexpression of nSMase2. PC12 cells were transfected with 1 μg plasmid-encoding V5-tagged nSMase2 or empty vector. **b** Reduced autophagic flux by knockdown of nSMase2. PC12 cells were transfected with 50 nM nSMase2 siRNA (si*Smpd3*) or non-targeting negative control siRNA (siControl). **c** Catalytically inactive mutants of nSMase2 were unable to induce autophagy. PC12 cells were transfected with either plasmid-encoding V5-tagged WT nSMase2, catalytically inactive forms of nSMase2 (D428A and H639A), or empty vector. At 48 h after transfection, the cells were incubated with HBSS or growth medium for 2 h. Autophagic flux was determined by the levels of LC3 puncta formation and p62 degradation. For the LC3 puncta counting assay of the overexpression group, LC3 puncta were counted in only V5-positive cells. For the LC3 turnover assay, transfected cells were starved with HBSS with or without 50 μM CQ for 2 h, and LC3 and GAPDH were analyzed by immunoblotting. LC3-II levels were normalized to GAPDH levels, and the quantified immunoblot assay data are presented as the mean ± SEM of at least three independent experiments. Scale bar = 5 μm; Significant differences between the indicated groups, *p < 0.05, **p < 0.01 and ***p < 0.001; NS, not significant

nSMase2 has been extensively studied among the neutral forms of SMase because of its actual and major physiological activity^28^. To examine the role of nSMase2 in starvation-induced autophagy, gene modulation experiments were conducted. Knockdown or overexpression of nSMase2 was confirmed by immunoblot analysis (Supplementary Figure 1B). Similar to nutrient starvation, overexpression of V5-tagged nSMase2 induced LC3 puncta formation, LC3 turnover, and degradation of the autophagy substrate p62 in V5-positive cells (Fig. 2a). Furthermore, the knockdown of *Smpd3*, which encodes nSMase2, by a pool of two small interfering RNAs (siRNAs) suppressed starvation-induced LC3 puncta formation, p62 degradation, and LC3 turnover (Fig. 2b). The inhibitory effect of nSMase2 knockdown on starvation-induced autophagic flux was also confirmed using the individual *Smpd3* siRNA and the additional pool of four other siRNAs (Supplementary Figure 2); inhibition was not due to off-target effects. The data imply that nSMase2 mediates starvation-induced autophagy.

GW4869 inhibits the enzyme activity of nSMase2^29^ and suppresses autophagy (Fig. 1), implying the requirement of nSMase2 activity for autophagy. To examine if the autophagy-inducing effects of nSMase2 involve its enzymatic activity, levels of autophagic flux were compared between cells expressing two catalytically inactive mutants (D428A, impaired substrate recognition; H639A, null in hydrolytic activity) and wild-type (WT) cells^8^. WT V5-tagged nSMase2 transfection induced autophagic flux as indicated by LC3 puncta formation, LC3 turnover, and p62 degradation. The induced flux was absent in both inactive mutants (Fig. 2c). nSMase2 overexpression in H639A, but not D428A, tended to decrease LC3 turnover compared to the vector transfected control, although the difference was not statistically significant. The finding was likely a dominant-negative effect of the H639A mutant, because the D428A mutant is defective in substrate binding, while H639A mutant that is impaired in hydrolytic activity could compete with endogenous nSMase2 for the binding of substrates. The absence of nSMase2 activity in the inactive mutants was confirmed by transiently overexpression (Supplementary Figure 3). These results suggest that nSMase2 may induce autophagy through its enzymatic activity.

### Starvation activates nSMase2 and increases ceramide formation in the Golgi apparatus

**Fig. 3:**
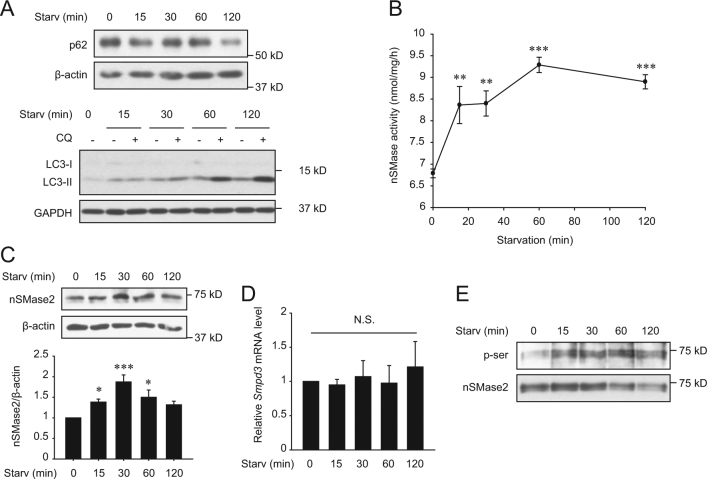
Nutrient starvation upregulates nSMase2. PC12 cells were starved with HBSS for the indicated times. **a** Induction of autophagy by nutrient starvation in PC12 cells. Degradation of p62 and LC3 turnover were detected by immunoblotting for assessing autophagic flux. For LC3 turnover assays, cells were starved with HBSS with or without 50 μM CQ for the indicated times. **b** Activation of nSMase2 by starvation. Specific activity of nSMase2 was analyzed using [^14^C]-labeled sphingomyelin. **c** Increase in nSMase2 expression by starvation. Protein expression levels of nSMase2 in starved cells were determined by immunoblotting and were normalized to β-actin levels. **d** No changes in nSMase2 mRNA levels were induced by starvation. The mRNA levels of nSMase2 were measured by quantitative real-time PCR and were normalized to *Hprt1*. **e** Starvation-induced phosphorylation of nSMase2 at a serine residue. PC12 cells were starved with HBSS for the indicated time, and cell lysates were incubated with biotin-conjugated sphingomyelin (the nSMase2 substrate) followed by pull-down with streptavidin-sepharose beads. The pellets were analyzed using immunoblots to detect serine phosphorylation of nSMase2. The data are presented as the mean ± SEM of three independent experiments. Significant differences, *p < 0.05, **p < 0.01, and ***p < 0.001 (one-way ANOVA followed by LSD test); NS, not significant

Considering the positive role of nSMase2 in autophagy induction, we next examined the activity or expression of nSMase2 during nutrient starvation. Both specific activity and protein expression of nSMase2 significantly increased under starvation, accompanied by autophagy, within 15–30 min after starvation (Fig. 3a–c). The rapid changes in nSMase2 expression suggested that regulation might occur at the post-transcriptional level, rather than the transcriptional level. As expected, mRNA levels of *Smpd3* were not altered during starvation (Fig. 3d). A previous study described enhanced protein stability and activity of nSMase2 by phosphorylation at multiple serine sites, which was regulated by protein phosphatase 2B (PP2B)^30^. Presently, the serine phosphorylation level of nSMase2 in starved cells was assessed by the nSMase2 pull-down assay. As expected, serine phosphorylation of nSMase2 increased during starvation (Fig. 3e).

**Fig. 4:**
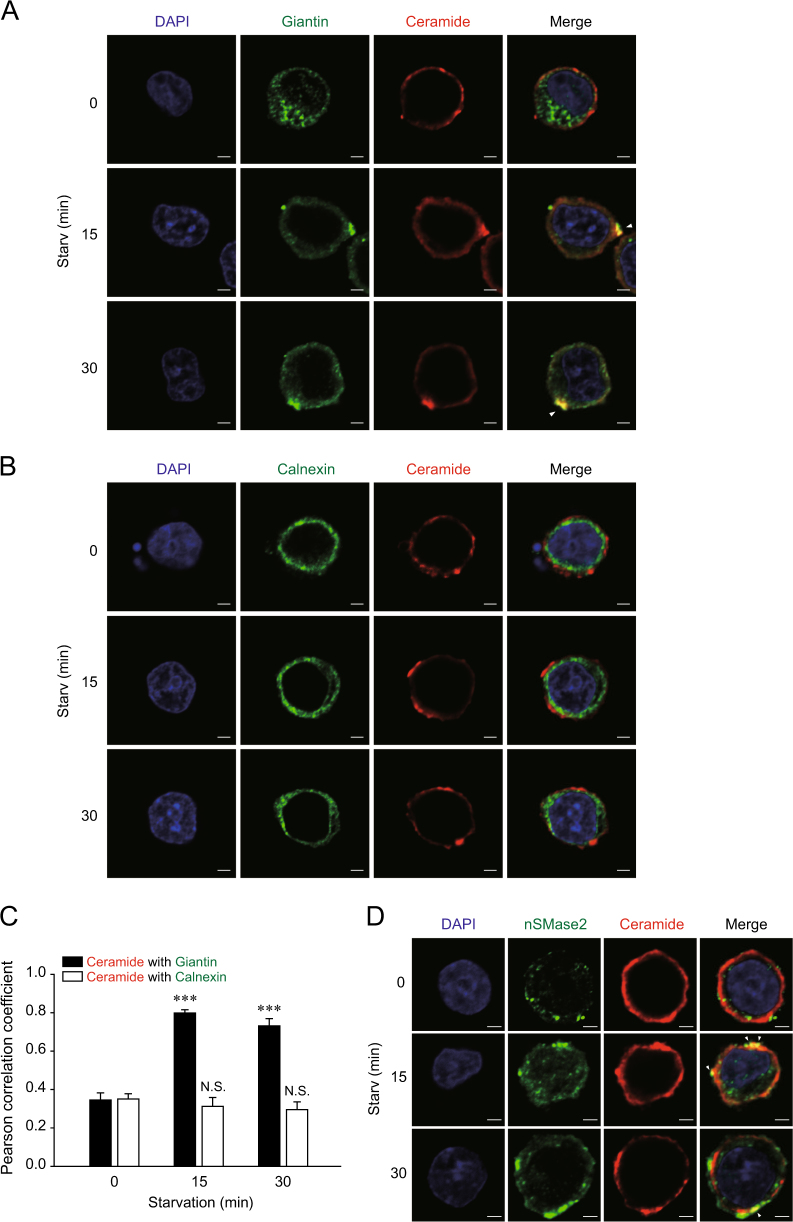
Nutrient starvation increases ceramide formation in the Golgi apparatus. PC12 cells were starved with HBSS for the indicated times. **a** Golgi-localized ceramides were observed during starvation. **b** No observable changes in ER-localized ceramide levels were evident during starvation. **c** The co-localization levels between ceramide and Golgi or ER marker were quantified using ImageJ software for more than 10 cells per each group. Significant differences, ***p < 0.001; NS, not significant **d** Increased co-localization of ceramide with nSMase2 is triggered by starvation. Starved cells were co-stained with antibodies against ceramide and the Golgi marker giantin, ER marker calnexin, or nSMase2. Yellow regions indicated by arrows in the merged images represent co-localization of green-stained nSMase2 or each organelle marker and red-stained ceramide. Scale bar = 2 μm

In cells, nSMase2 is mainly localized to the Golgi apparatus and plasma membrane^8,9^, while CerS is localized to the ER^7^. Thus, localization of ceramides in nutrient-starved cells was assessed by immunofluorescence (IF). During nutrient starvation with HBSS, increased co-localization of ceramides and giantin (the Golgi marker) was observed (Fig. 4a). However, there were no observable changes in ER-localized ceramide levels (Fig. 4b). The co-localization of ceramides and Golgi or ER marker were quantified using Pearson’s correlation coefficient, with 1 and 0 indicating perfect and no correlation, respectively (Fig. 4c). In addition, co-localization of ceramides and nSMase2 was induced by starvation (Fig. 4d). The specificity of antibody binding to nSMase2 during IF assays was confirmed in V5-tagged nSMase2-overexpressing cells (Supplementary Figure 4).

### Inhibition of ceramide transfer protein (CERT) or sphingomyelin synthase (SMS) represses starvation-induced autophagic flux

**Fig. 5:**
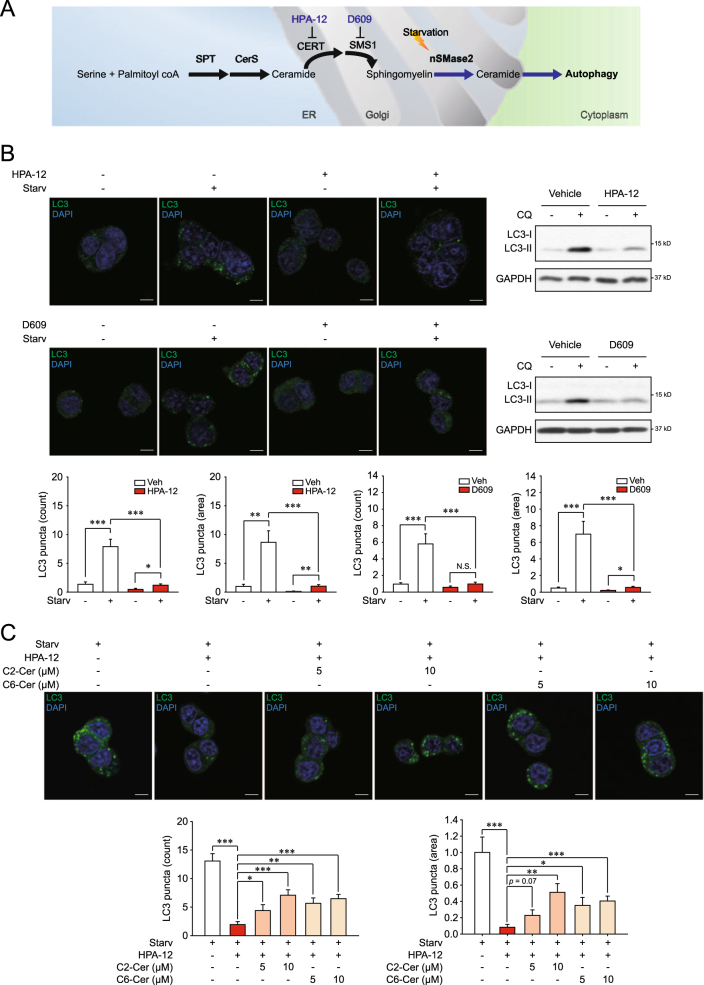
HPA-12 and D609 suppresses starvation-induced autophagy. **a** A schematic diagram illustrating the proposed model in which the enzymatic reaction by nSMase2 is a key step in regulating autophagy. **b** Decreased autophagic flux by either CERT or SMS inhibition. PC12 cells were pretreated with 10 μM HPA-12, 100 μM D609 or the vehicle for 1 h and then starved for 2 h in the presence or absence of 50 μM CQ and 10 μM HPA or 100 μM D609. LC3 puncta formation and LC3 turnover were analyzed to determine autophagic flux. **c** Recovery of CERT inhibition-attenuated autophagic flux by ceramide supply. PC12 cells were pretreated with 10 μM HPA-12 or the vehicle for 1 h and then starved for 2 h with C2-ceramide or C6-ceramide at the indicated concentrations in the presence or absence of 10 μM HPA-12. Autophagic flux was measured by LC3 puncta formation. Scale bar = 5 μm; Significant differences between the indicated groups, *p < 0.05, **p < 0.01, and ***p < 0.001; NS, not significant

To evaluate if localization of ceramides to the Golgi complex is required to trigger autophagy, we examined the effects of a CERT inhibitor (HPA-12) and an SMS inhibitor (D609) on starvation-induced autophagic flux. As depicted in Fig. 5a, CERT transfers the de novo-synthesized ceramide from the ER to the Golgi complex^31^, followed by the synthesis of SM by SMS1 using the transferred ceramide^32^. HPA-12 and D609 limited the formation of SM by retaining ceramides in the ER, preventing their use by nSMase2^33,34^. As expected, treatment with HPA-12 or D609 significantly reduced the starvation-induced autophagic flux (Fig. 5b).

We also checked whether the ceramide supply could rescue the suppressed autophagic flux by inhibition of ceramide transport from the ER to the Golgi complex. Treatment of the C2- and C6-ceramides that reach Golgi complex^35^ significantly increased the autophagic flux inhibited by HPA-12 (Fig. 5c). The data imply that the Golgi-localized ceramide is required for induction of autophagy. Collectively, these results support the notion that production of ceramides by nSMase2 in the Golgi apparatus plays a key role in autophagy.

### nSMase2-mediated induction of autophagy occurs via activation of p38 MAPK and inhibition of mTOR

**Fig. 6:**
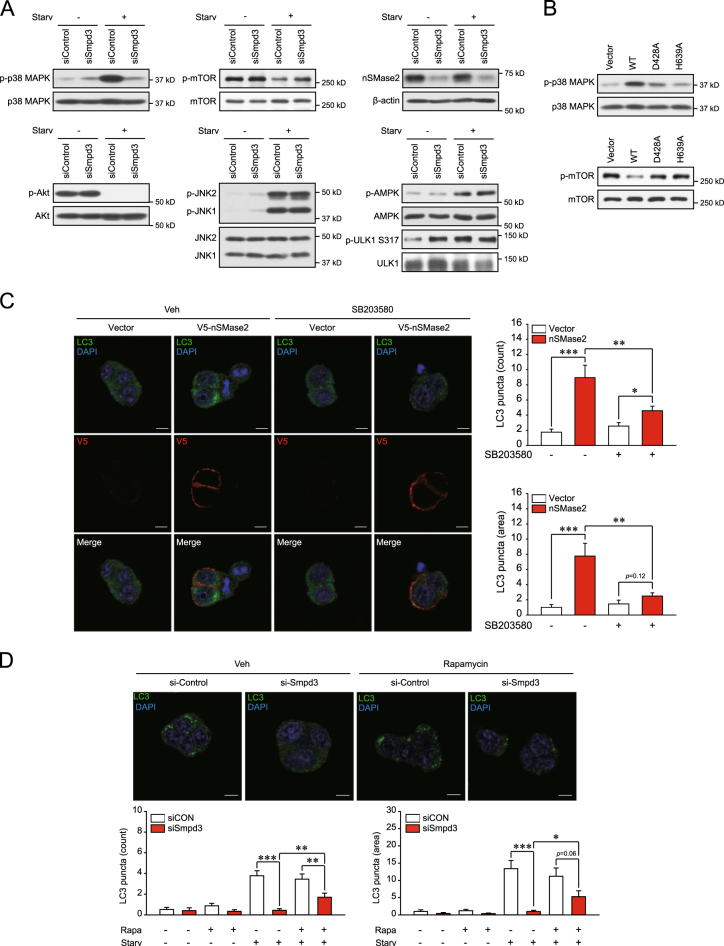
nSMase2 induces autophagy via p38 MAPK activation and mTOR inhibition. **a** Suppression of starvation-induced p38 MAPK phosphorylation and starvation-reduced mTOR phosphorylation by knockdown of nSMase2. PC12 cells were transfected with 50 nM nSMase2 siRNA (si*Smpd3*) or non-targeting negative control siRNA (siControl). At 48 h after transfection, the cells were incubated with HBSS for 2 h. Phospho-p38 MAPK, p38 MAPK, phosphor-mTOR, mTOR, phospho-Akt, Akt, phospho-JNK1/2, JNK1/2, phospho-AMPK, AMPK, phospho-ULK1 (Ser317), and ULK1 levels were analyzed by immunoblotting, and nSMase2 knockdown was confirmed simultaneously by immunoblotting. **b** Enhanced p38 MAPK phosphorylation and suppressed mTOR phosphorylation by nSMase2. PC12 cells were transfected with either plasmid-encoding V5-tagged WT nSMase2, catalytically inactive forms of nSMase2 (D428A and H639A), or empty vector. At 48 h after transfection, phospho-p38 MAPK, p38 MAPK, phospho-mTOR, and mTOR levels were analyzed by immunoblotting. **c** p38 MAPK inhibition with SB203580 attenuated the autophagy induction by nSMase2 overexpression. Cells overexpressing V5-tagged nSMase2 were pretreated with 10 μM SB203580 or the vehicle for 3 h. LC3 puncta were detected by immunofluorescence assay using anti-LC3 antibody and counted using ImageJ software in V5-positive cells. Scale bar = 5 μm. **d** mTOR inhibition with rapamycin partially reversed the effect of nSMase2 knockdown on LC3 puncta formation. PC12 cells transfected with siRNA (as described above) were pretreated with 750 nM rapamycin or the vehicle for 1 h and then starved for 2 h. LC3 puncta were detected by immunofluorescence assay using anti-LC3 antibody and counted using ImageJ software. Representative images of siRNA-transfected cells under starvation conditions are provided. Scale bar = 5 μm; Significant differences between the indicated groups, *p < 0.05, **p < 0.01, and ***p < 0.001

To explore the downstream pathways of nSMase2 involved in autophagy regulation, we examined the effects of nSMase2 on phosphorylation of p38 mitogen-activated protein kinase (MAPK), mechanistic target of rapamycin (mTOR), Akt, c-Jun N-terminal kinase (JNK) 1/2, AMP-activated protein kinase (AMPK), and Unc-51 like autophagy activating kinase (ULK1). All are signaling molecules reported to regulate the autophagy machinery. The Akt-mTOR pathway is a crucial negative regulator of autophagy^36^, while the JNK-beclin 1 and the AMPK-ULK1 pathways are positive regulators^37,38^. Although the role of p38 MAPK in autophagy is still controversial, one study reported starvation-induced autophagy mediated by p38 MAPK^39^. Phosphorylation of p38 MAPK increased during starvation in HBSS, and starvation-induced p38 MAPK phosphorylation was reduced by nSMase2 knockdown (Fig. 6a). Akt is known as an upstream activator of mTOR^40^, and ceramides are regarded as mTOR regulators via Akt inhibition^41^. However, starvation-induced reduction of mTOR phosphorylation was suppressed by siRNA knockdown of nSMase2, without a decrease in Akt phosphorylation (Fig. 6a). The phosphorylation level of Akt was unaltered, even by nSMase2 overexpression (Supplementary Figure 5A). JNK1/2 is also modulated by ceramide treatment^16^, but starvation-induced JNK1/2 phosphorylation was not suppressed by transfection with nSMase2 siRNA (Fig. 6a). AMPK direct activates ULK1 by phosphorylating Ser317, which promotes autophagy^38^, and phosphorylation of AMPK is affected by ceramide treatment^42–44^. However, the enhanced phorphorylation levels of AMPK and ULK1 (Ser 317) by starvation were not reduced by nSMase2 knockdown, while the transfection with nSMase2 siRNA rather increased phosphorylation of ULK1 at Ser 317 in the basal nutrition condition (Fig. 6a). Additionally, overexpression of nSMase2 enhanced p38 MAPK phosphorylation and reduced mTOR phosphorylation (Fig. 6b). Notably, the effects of wild-type nSMase2 overexpression on p38 MAPK and mTOR phosphorylation were not detected for the enzymatically inactive mutants of nSMase2 (Fig. 6b). The data indicate that nSMase2 activates p38 MAPK and inhibits mTOR signaling.

p38 MAPK inhibition by SB203580 suppressed the LC3 puncta formation triggered by nSMase2 overexpression (Fig. 6c). Furthermore, the nSMase2-induced p62 degradation was repressed by inhibition of p38 MAPK (Supplementary Figure 5C). Rapamycin-mediated inhibition of mTOR partially reversed the effect of nSMase2 knockdown on LC3 puncta formation during starvation (Fig. 6d), although this reversal effect of rapamycin was not observed in the p62 degradation assay (Supplementary Figure 5D). The inhibitory effects of SB203580 and rapamycin on p38 MAPK and mTOR, respectively, were valid at the concentrations used in the experiments (Supplementary Figure 5B). Together, these results indicate that nSMase2-mediated induction of autophagy occurs through signaling pathways via p38 MAPK activation and mTOR inhibition.

### nSMase2 plays a protective role against toxic stress depending on autophagy and its levels are reduced in PD

**Fig. 7:**
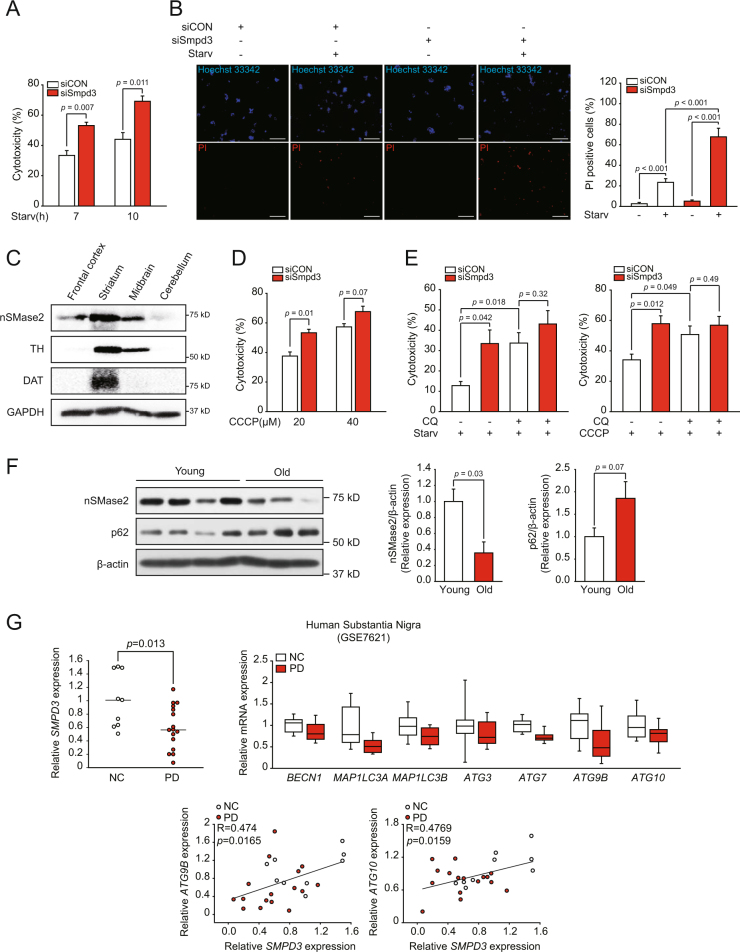
Cytoprotective role of nSMase2 against starvation and CCCP exposure depending on autophagy, and its relationship with PD. **a** Pro-survival effect of nSMase2 against starvation. PC12 cells were transfected with 50 nM nSMase2 siRNA (si*Smpd3*) or non-targeting negative control siRNA (siCON). At 48 h after transfection, cells were incubated with HBSS for 7 or 10 h. Levels of lactate dehydrogenase (LDH) released into the medium and total LDH were quantified to calculate cell cytotoxicity. The data represent the mean ± SEM of three independent experiments. **b** Images of dead cells. PC12 cells transfected with siRNA were starved for 10 h and then double-stained with PI and Hoechst 33342. Dead cells were positive for PI, and total cells were labeled with Hoechst 33342. PI positive cells were counted for more than 800 cells per group. Scale bar = 100 μm **c** High expression of nSMase2 in the dopaminergic neuron-enriched area. Expression level of nSMase2 was detected in the mouse brain by immunoblotting. **d** Cytoprotective effect of nSMase2 against CCCP exposure. PC12 cells transfected with siRNA were treated with 20 or 40 μM CCCP for 24 h. **e** Autophagy-dependent cytoprotective role of nSMase2. The cells transfected with indicated siRNA were starved for 7 h or treated with 20 μM CCCP for 24 h in the presence or absence of 50 μM CQ. Cytotoxicity was determined by the LDH assay. The data represent the mean ± SEM of three independent experiments. **f** Reduced levels of nSMase2 in the striatum of old mice with p62 accumulation. Expression levels of nSMase2 and p62 in young (6 weeks) and old (20 months) mice were assessed by immunoblotting and normalized to β-actin levels. **g** Decreased expression of nSMase2 and autophagy-associated genes in the substantia nigra of patients with PD (upper). Positive correlation between nSMase2 and ATGs (lower). Data were extracted from GSE7621 data set (Control, *n* = 9; PD patients, *n* = 16)

Since autophagy primarily acts as a survival mechanism against various stressors, we examined if nSMase2 has a protective function in stress-induced cytotoxicity through induction of autophagy. Starvation-induced cell death, measured by lactate dehydrogenase (LDH) release, increased after nSMase2 knockdown (Fig. 7a). Moreover, the number of propidium iodide (PI)-positive (dead) cells also increased during starvation after nSMase2 knockdown, indicating a cytoprotective role for nSMase2 (Fig. 7b). nSMase2 is highly expressed in the brain and bones^20^. Presently, nSMase2 was highly expressed in mouse striatum and midbrain, including the substantia nigra region, as verified by the enrichment of the dopaminergic neuron markers tyrosine hydroxylase and dopamine transporter (Fig. 7c). This finding prompted us to examine dopaminergic stress-induced cytotoxicity. Because mitochondrial dysfunction plays a central role in pathogenesis of PD^45^, the cytotoxicity induced by the mitochondrial uncoupler carbonyl cyanide m-chlorophenyl hydrazine (CCCP) was tested. nSMase2 knockdown significantly increased CCCP-induced cytotoxicity (Fig. 7d). Furthermore, CCCP-induced autophagic flux was attenuated by nSMase2 knockdown or inhibition (Supplementary Figures 6A and 6B), demonstrating that nSMase2 contributes to CCCP-induced autophagy. In addition to mitochondrial dysfunction, oxidative stress and protein adducts caused by dopamine oxidation can contribute to the progression of PD^46^. Thus, we examined the effect of a high dose of dopamine exposure. Consistent with the CCCP treatment results, nSMase2 knockdown enhanced the cell death induced by the high dose of dopamine (Supplementary Figure 6C). The results indicate the protective role of nSMase2 against dopaminergic toxicity.

To confirm whether the cell-protective role of nSMase2 depends on autophagy regulation, cytotoxicity was determined in the presence or absence of the autophagy inhibitor CQ. There were no significant differences in starvation or CCCP-induced cytotoxicity between siControl and si*Smpd3* transfected cells in the presence of CQ, demonstrating that autophagy inhibition is epistatic to nSMase2 repression in cell death (Fig. 7e). Thus, the cytoprotective role of nSMase2 depends on autophagy. Treatment with CQ enhanced cell death induced by starvation or CCCP treatment in control siRNA transfected cells (Fig. 7e), also implying that cells experiencing the toxic stress activate the autophagy process as a protective mechanism.

One of the major risk factors for PD is aging, which may reduce the autophagic function. Thus, we examined the level of nSMase2 in the brain, especially in the striatum, of old and young mice. As expected, the level of nSMase2 expression was lower and p62 accumulation was higher in old mice than in young mice (Fig. 7f). Furthermore, we analyzed the correlation between nSMase2 and PD using a GEO database (GSE7621) of human substantia nigra. Interestingly, *SMPD3* expression in the patient group was significantly lower than in the healthy group (Fig. 7g). The expression of some autophagy-associated genes was also reduced in patients with PD, and significant correlations between *SMPD3* and *ATG* genes, such as *ATG9B* and *ATG10*, were observed (Fig. 7g).

**Fig. 8:**
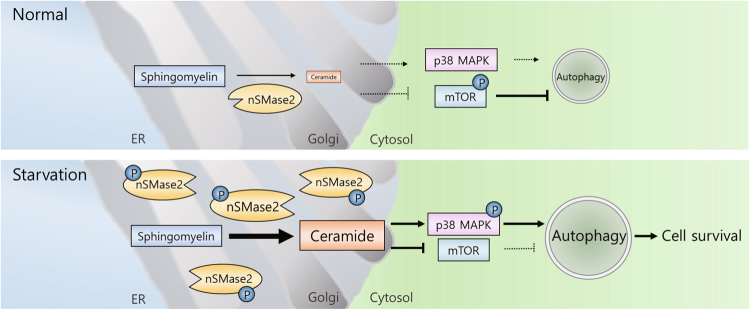
Working model for the nSMase2-mediated induction of cytoprotective autophagy during starvation. Under normal conditions, autophagy is suppressed by phosphorylated mTOR. During nutrient starvation, upregulated nSMase2 produces ceramides in the Golgi that activate p38 MAPK and inhibits mTOR signaling, thereby inducing autophagic flux

The collective findings demonstrate the nSMase2-mediated induction of cytoprotective autophagy via p38 MAPK activation and mTOR suppression (Fig. 8), and strongly suggest the preventive or therapeutic role of nSMase2 for PD through autophagy induction.

## Discussion

Autophagic processes are closely associated with membrane dynamics^1,5^. Ceramides play central roles in sphingolipid metabolism as membrane components that mediate membrane dynamics by altering fluidity or rigidity, and can also function as signaling molecules^6^. In this context, several studies have revealed that treatment with exogenous ceramides can induce autophagy^16–18,47^. Recently, the de novo pathway of ceramide synthesis was suggested to be involved in melatonin-induced autophagic cell death^48^ and sodium selenite-induced lethal mitophagy^49^. In contrast, this study showed that in a nutrient starvation model, arguably the most physiologically relevant stimulus of autophagy^50^, the de novo pathway of ceramide synthesis had negligible effect on autophagic flux (Fig. 1). Moreover, nSMase2 knockdown or inhibition with a specific inhibitor significantly suppressed starvation-induced autophagic flux (Figs. 1, 2), suggesting that nSMase2 mediates starvation-induced autophagy.

Increased serine phosphorylation of nSMase2 can enhance protein stability^30^. Consistent with this, we found that under nutrient-starvation conditions, nSMase2 protein level and activity rapidly and stably increased within 15–30 min. nSMase2 was phosphorylated at serine residues within 15 min after starvation, with no change in mRNA levels, with subsequent LC3 turnover and p62 degradation within 60–120 min (Fig. 3). This suggests that activation of nSMase2 can lead to the induction of autophagy. We also found that treatment with cyclosporine A (CsA), a PP2B inhibitor, had a similar effect on autophagy as the overexpression of nSMase2 (Supplementary Figure 7). A study on cardiomyocytes also supported our finding^51^. Thus, nutrient starvation may inhibit dephosphorylation of nSMase2 by PP2B, thereby inducing nSMase2-mediated autophagy.

In particular, the Golgi-localized ceramides induced by starvation (Fig. 4) and the diminished autophagy by CERT inhibitor that limited ceramide transport from the ER to the Golgi complex, which was recovered by ceramide supply (Fig. 5), suggest that localization of ceramides to the Golgi complex is the key step of autophagy regulation, with respect to the flow of ceramides. Inhibition of CERT and SMS, which are the upstream molecules of nSMase2, suppressed autophagy, while the inhibition of de novo synthesis upstream of CERT did not affect autophagy during starvation. The reason might be the sufficient basal level of ceramide in ER. The markedly greater level of ceramides in ER than in the Golgi apparatus^52^ indicates that the level of Golgi-localized ceramides reply mainly on the direct supply to the Golgi, rather than on the de novo synthesis of ceramide in the ER.

To elucidate the mechanism of starvation-induced autophagy via the activation of the nSMase2-ceramide pathway in the Golgi complex, we examined the correlation between activation of this pathway and several regulatory pathways of autophagy. In one study, p38 MAPK mediated starvation-induced autophagy in mouse embryonic fibroblasts^39^. However, the role of p38 MAPK in autophagy remains largely unknown. Consistent with the previous study, we found that starvation strongly induced phosphorylation of p38 MAPK (Fig. 6a). While p38 MAPK can activate nSMase2^53,54^, our results instead showed that nSMase2 can mediate the phosphorylation of p38 MAPK induced by starvation (Fig. 6a). This phosphorylation was significantly reduced by knockdown or enzymatic inactivation of nSMase2. Moreover, treatment with the p38 MAPK inhibitor, SB203580, diminished nSMase2 overexpression-induced LC3 puncta formation and p62 degradation (Fig. 6c and Supplementary Figure 5C), suggesting that nSMase2-induced phosphorylation of p38 MAPK explains their sequential roles in autophagy.

Our data also suggest that nSMase2 inhibition of mTOR is involved in nSMase2-dependent autophagy induction during starvation (Fig. 6a–d). Inhibition of Akt may be the cause of mTOR inhibition by nSMase2, because ceramides inhibit Akt, and Akt activates mTOR^40,41^; however, Akt phosphorylation was not affected by knockdown of nSMase2 (Fig. 6a), indicating that the inhibition of mTOR cannot be explained by ceramide-induced inhibition of Akt. Similarly, ceramides can activate the JNK-beclin 1 pathway^16^. However, nSMase2 knockdown did not affect JNK (Fig. 6a). Although the effect of ceramide on AMPK is still controversial, ceramide-mediated induction of AMPK phosphorylation was described^42^. However, starvation-induced phosphorylation of AMPK and its downstream target ULK1 at serine 317 were not reduced by nSMase2 knockdown (Fig. 6a). Since the increased phosphorylation of ULK1 at the serine 317 residue by nSMase2 knockdown occurred only in the basal condition without starvation and was not accompanied by AMPK activation, the effect of nSMase2 knockdown on ULK1 was not studied further, which would be elucidated by later studies. The precise mechanism by which nSMase2-derived ceramides in the Golgi complex regulate p38MAPK and mTOR as key effector molecules in autophagic processes also remains to be elucidated. Nevertheless, these results indicate that ceramides can mediate autophagy through starvation-induced activation of nSMase2, and is related to both p38 MAPK activation and mTOR inhibition as a possible signaling molecule.

Additionally, the mechanical role of ceramides in membrane dynamics may be a possible mechanism of nSMase2-mediated induction of autophagy. This needs to be investigated. Conversion of SM to ceramides by bacterial SMase induces lipid membrane modifications, such as vesicle collapse and rupture^55^. Moreover, in mammalian cells, nSMase2 deficiency disturbs the Golgi secretory pathway^56^ and exosome budding into multivesicular endosomes^23,24^. During autophagy, cellular organelles including the Golgi apparatus^1,57^ participate in autophagosome formation by providing membrane sources. The inhibition of membrane supply from the Golgi results in failure of vesicle formation during autophagy^58^. Further investigation regarding nSMase2 regulation of Golgi membrane dynamics, such as budding to provide membrane sources for autophagosomes, may contribute to a better understanding of how nSMase2 induces autophagic flux.

Autophagy primarily plays a vital role in maintaining cellular homeostasis that prolongs cell survival, but lethal autophagy also exists. Lethal autophagy is induced by exogenous ceramide^16,17^ and CerS^48,49^. In contrast, nSMase2 can be cytoprotective during starvation (Fig. 7a,b), implying that the survival or lethal outcome of ceramide-induced autophagy may depend on its subcellular localization and role in signaling and/or membrane dynamics. Highly secretory cells, such as neurons, may be more dependent on cytoprotective autophagy because of the accumulation of higher levels of misfolded proteins and aggregates following abundant protein synthesis. Protein aggregation is a common feature of various neurodegenerative diseases, including PD and AD^4^, and depletion of *ATG* causes neurodegeneration^59^. nSMase2 is highly expressed in brain^9^. In the brain, nSMase2 was abundant in the striatum and midbrain (Fig. 7c) containing dopaminergic neurons which are lost in PD^60^. nSMase2 also protected cells against dopaminergic stressors (Fig. 7d and Supplementary Figure 6C). The cytoprotective role of nSMase2 may depend on autophagy (Fig. 7e).

In addition, as shown in Fig. 7f, g, nSMase2 gene expression in the substantia nigra of patients with PD was significantly reduced compared with healthy controls, and protein expression of nSMase2 in the mouse striatum decreased with aging—a major risk factor for PD^61^. Consistent with our finding, in a neurodegeneration model of *Drosophila* with defects in autophagy, neutral SMase overexpression rescued neurodegeneration^62^. Ciliogenesis and differentiation for neural development mediated by nSMase2 were reported in human stem cells and neural progenitor cells^63^. On the other hand, nSMase2 deficiency influenced recovery the 5XFAD mouse model of early onset AD with amyloid-β pathology^64^. However, the model does not seem to fully reflect human AD because of the lack of taupathy^65^, which is another major pathological hallmark and a more potent target of AD in human clinical trials^66^. In addition, in contrast to other AD models including the human tau transgenic mouse model, in which autophagy activation alleviates the disease state^67,68^, the inhibition of autophagy can ameliorate AD pathology in the 5XFAD model^69^. Therefore, the effect of nSMase2 modulation in other AD models, including the taupathy model, could provide clearer understanding of its action on AD. Indeed, inhibition of nSMase2 disrupts synaptic plasticity and memory in C57BL/6 mice ^70^.

This study demonstrates that nSMase2 can mediate starvation-induced autophagy and that activation of nSMase2 is essential for autophagy induction. This suggests that the nSMase2-Golgi-localized ceramide pathway plays a critical role in autophagy, providing insights into the mechanisms underlying human diseases.

## Experimental procedures

### Reagents

RPMI-1640 medium, horse serum (HS), fetal bovine serum (FBS), HBSS, phosphate-buffered saline (PBS), penicillin/streptomycin, Lipofectamine RNAiMAX, Lipofectamine 2000, 4’,6-diamidino-2-phenylindole (DAPI), ProLong Gold, Alexa-conjugated secondary antibodies (Alexa Fluor 488-conjugated goat anti-rabbit IgG, A11008; Alexa Fluor 594-conjugated goat anti-mouse IgM, A-21044; Alexa Fluor 594-conjugated goat anti-mouse IgG, A-11032), and anti-V5 antibody (#46-0705) were purchased from Thermo Fisher Scientific (Waltham, MA, USA). Poly-D-lysine-coated 6-well plates, 24-well plates, and 100-mm dishes were obtained from Corning (Corning, NY, USA). Poly-D-lysine-coated coverslips (GG-12-PDL) were supplied by Neuvitro (Vancouver, WA, USA). Desipramine, FB1, and SB203580 were obtained from Tocris Bioscience (Bristol, UK). GW4869, myriocin, and anti-ULK1 antibody (A7481) were purchased from Sigma-Aldrich (St. Louis, MO, USA). HPA-12 was supplied by Tokyo Chemical Industry (Tokyo, Japan). Protease inhibitor cocktail and PhosSTOP phosphatase inhibitor cocktail were provided by Roche (Basel, Switzerland). Bovine serum albumin (BSA) was purchased from Bioworld (Dublin, OH, USA). Anti-LC3B (NB100-2220) and anti-p62 antibodies (H00008878-M01) were obtained from Novous Biologicals (Littleton, CO, USA). C2-ceramide, C6-ceramide, anti-ceramide mouse IgM (ALX-804-196-T050), and anti-calnexin antibody (ADI-SPA-860-D) were provided by Enzo Life Sciences (Farmingdale, NY, USA). Anti-giantin (ab24586) antibody was obtained from Abcam (Cambridge, UK). Anti-phospho-p38 MAPK (Thr180/Tyr182; #4631), anti-p38 MAPK (#9212), anti-phospho-mTOR (Ser2448; #2971), anti-mTOR (#2972), anti-phospho-Akt (Ser473; #4058), anti-Akt(#4685), anti-phospho-JNK1/2 (Thr183/Tyr185; #4668), anti-JNK1/2 (#9252), anti-phospho-AMPKα (Thr172; #2535), anti-AMPKα (#2532), anti-phospho-ULK1 (Ser317; #12753), and horseradish peroxidase (HRP)-conjugated secondary antibodies (anti-rabbit IgG, #7074; anti-mouse IgG, #7076) were purchased from Cell Signaling Technology (Danvers, MA, USA). Anti-nSMase2 (sc-166637 for immunoblot analysis; sc-67305 for immunofluorescence analysis), anti-tyrosine hydroxylase (sc-14007), anti-glyceraldehyde 3-phosphate dehydrogenase (GAPDH, sc-25778), and anti-β-actin (sc-47778) antibodies were provided by Santa Cruz Biotechnology (Santa Cruz, CA, USA). Anti-phosphoserine (AB1603) and anti-dopamine transporter (MAB369) antibodies were obtained from EMD Millipore (Billerica, MA, USA). Dimethyl sulfoxide (DMSO) was supplied by Duchefa Biochemie (Haarlem, Netherlands). Unless otherwise stated, all other reagents were purchased from Sigma-Aldrich.

### Cell culture and treatment

Rat neuroblastoma PC12 cell line was purchased from American Type Culture Collection (Manassas, VA, USA). PC12 cells were cultured in RPMI-1640 medium containing 10 % HS and 5% FBS at 37 °C in a humidified atmosphere of 5 % CO_2_. For nutrient starvation, cells were incubated with HBSS for 2 h in the presence or absence of 50 μM CQ after washing with HBSS. For inhibitor studies, the indicated concentration of each inhibitor or vehicle was added to cells 1 h prior to and during the starvation period.

### Transfection with siRNA or plasmid

For nSMase2 knockout, a pool of two siRNAs against rat *Smpd3* (RSS331830 and RSS331831, Stealth siRNA, Thermo Fisher Scientific) was used throughout the study with negative control siRNA (#12935-300) as a control. ON-TARGETplus SMART pool siRNA of rat *Smpd3* (L-095934-02; Dharmacon) and its negative control siRNA (D-001810-10) were additionally used in the experiment confirming the effect of nSMase2 knockdown on autophagy. PC12 cells were transfected with 50 nM of the indicated siRNA using Lipofectamine RNAiMAX. For overexpression of V5-tagged nSMase2, cells were transfected with pcDNA3.1/V5-His-TOPO vector encoding murine *Smpd3* using Lipofectamine 2000 following manufacturer’s instructions. The vector was a gift from Dr. Yusuf Hannun. Cells were used for further experiments at 48 h post-transfection.

### Immunoblot analysis

Cells were homogenized by sonication in radioimmunoprecipitation assay (RIPA) buffer (Bioworld) containing protease inhibitor cocktail and PhosSTOP phosphatase inhibitor cocktail. The lysates were centrifuged at 10,000 × *g* at 4 °C for 10 min, and the supernatants were collected. Proteins in the lysates were separated by sodium dodecyl sulfate-polyacrylamide gel electrophoresis (SDS-PAGE) and transferred to a polyvinylidene fluoride membrane (Bio-Rad Laboratories, Hercules, CA, USA). The membranes were blocked for 1 h using Tris-buffered saline (TBS) containing 5 % BSA and 0.1% Tween-20, and then incubated with primary antibodies against target proteins in 5% BSA and 0.1% Tween-20 in TBS for 24 h at 4 °C. After three washes with 0.1% Tween-20 in TBS, the membranes were incubated with HRP-conjugated secondary antibodies in 5% BSA and 0.1% Tween-20 in TBS for 1 h at room temperature (RT). Proteins were detected using the chemical reaction of HRP with electrochemiluminescence (ECL) substrates (GenDEPOT, Katy, TX, USA).

### Immunofluorescence assay

Cells were fixed with 4 % formaldehyde in PBS for 20 min. Fixed cells were washed three times with 0.3% Triton X-100 (TX-100) in PBS and incubated with 5% BSA and 0.3% TX-100 in PBS for 1 h at RT. The cells were then incubated with primary antibodies (anti-ceramide, 1:10; anti-nSMase2, 1:100; anti-LC3, 1:300; anti-V5, 1:500; anti-giantin, 1:500; anti-calnexin, 1:500) against target proteins in 1% BSA and 0.3% TX-100 in PBS for 24 h at 4 °C. After three washes, cells were incubated with Alexa-conjugated secondary antibodies (1:350) for 1 h at RT. DAPI was used for nuclear staining and coverslips were mounted with ProLong Gold antifade reagent after three washes. Cell images were acquired with a confocal microscope (LSM 710; Zeiss, Oberkochen, Germany). LC3-positive puncta numbers and size were measured with the GFP-LC3 macro^71^ using ImageJ software (NIH, Bethesda, MD, USA) for more than 15 cells per group.

### nSMase2 activity assay

nSMase2 activities of 10 μg of cell homogenates were assessed using [^14^C]-sphingomyelin as a substrate in a mixture with 100 mM Tris-HCl (pH 7.5) buffer containing 0.1% TX-100, 10 mM MgCl_2_, 5 mM dithiothreitol, and 50 μM phosphatidylserine as described previously^21^. The reaction proceeded for 20 min at 37 °C and terminated by adding chloroform/methanol/2N HCl (1:1:0.1, v/v). Samples were centrifuged for phase separation, and the radioactivity of the upper phase was determined by liquid scintillation counting (MicroBeta2; PerkinElmer, Waltham, MA).

### Pull-down of nSMase2

For pull-down of nSMase2, 30 μg of cell lysates in 100 mM Tris-HCl (pH 7.5) buffer containing 0.1% Triton X-100 and 10 mM MgCl_2_ were incubated with 10 μM biotin-conjugated SM (Echelon, S-400B) on a rocker for 2 h at 4 °C. Sequentially, streptavidin-Sepharose beads (GE Healthcare, Buckinghamshire, UK, #71-5004-40 AE) was added to the samples and incubated on a rocker overnight at 4 °C. The samples were centrifuged at 700 × *g* at 4 °C for 1 min, and the collected pellets were washed five times with cold PBS. After washing, the pellets were treated with SDS sample buffer and continuously analyzed by immunoblotting.

### Detection of mRNA levels

RNA was extracted from PC12 cells using TRIzol reagent (Invitrogen, Carlsbad, CA, USA). For cDNA synthesis, the extracted RNA was reverse transcribed using a SuperScript III First-Strand Synthesis System (Invitrogen). Quantitative real-time polymerase chain reaction (PCR) was performed using SYBR Green Supermix (Bio-Rad Laboratories) and detected by the CFX Connect™ real-time PCR detection system (Bio-Rad Laboratories) according to the manufacturer’s instructions. mRNA expression was normalized to *Hprt1* mRNA levels.

To confirm the expression of ceramide-producing enzymes in PC12 cells, extracted mRNA was analyzed by reverse transcription PCR (RT-PCR). After cDNA synthesis was performed as described above, Maxime i-Taq PCR PreMix (iNtRON Biotechnology, Seongnam, Republic of Korea) was used for PCR. PCR products were detected using the ChemiDoc imaging system (Bio-Rad Laboratories) after PAGE. All PCR primers used in this study are listed in Supplementary Table 1.

### LDH assay

Cell cytotoxicity was determined by LDH release using the CytoTox 96^®^ Non-Radioactive Cytotoxicity Assay (G1780; Promega, Madison, WI, USA) according to the manufacturer’s instructions. Cells cultured in poly-d-lysine-coated 24-well plates (1.5 × 10^5^ cells/well) were subjected to nutrient starvation and treated with the indicated concentrations of dopamine or CCCP. After the designated treatment times, culture supernatants were collected and incubated with the reaction mixture to measure LDH activity. LDH released into the medium was calculated as the percentage of total LDH.

### PI/Hoechst 33342 stain

To image dead cells, PC12 cells were seeded on poly-D-lysine-coated coverslips (1.5 × 10^5^ cells/well) and cultured in growth medium for 1 day. Cells under the indicated stress conditions were incubated with 50 ng/mL PI (BD Biosciences, #51-66211E) and 10 μg/mL Hoechst 33342 dye (Thermo Fisher Scientific, H3570) for 30 min. Images of cells stained with blue-fluorescent Hoechst 33342 and red-fluorescent PI were obtained by a Ni-U fluorescence microscope (Nikon, Tokyo, Japan) equipped with a digital camera (DS-Ri1; Nikon).

### Brain dissection

Young (6 weeks) and old (20 months) male C57BL/6 mice were obtained from the Korea Research Institute of Bioscience and Biotechnology (Daejeon, Korea). All animal experiments were performed in accordance with the National Research Council’s Guidelines for the Care and Use of Laboratory Animals and Guidelines for Animal Experiments of Chung-Ang University, and were approved by the University Committee for Animal Experiments (approval no: 2014-00031). Mice brains were dissected according to a previously described method^72^, and the brain tissues were analyzed by immunoblotting.

### Statistical analysis

All data are expressed as the mean ± standard error of the mean (SEM). Unless otherwise stated, differences between two groups were analyzed using the Student’s *t*-test. In all cases, a p-value < 0.05 was considered statistically significant.

## Electronic supplementary material

Figure S1

Figure S2

Figure S3

Figure S4

Figure S5

Figure S6

Figure S7

Table S1

Supplementary figure legends
