# Supplementary figures and images for: Activation of neutral sphingomyelinase 2 by starvation induces cell-protective autophagy via an increase in Golgi-localized ceramide
Source: Cell Death Dis. 2018 Jun 4;9(6):670. doi: 10.1038/s41419-018-0709-4 (PMC5986760; doi:10.1038/s41419-018-0709-4)

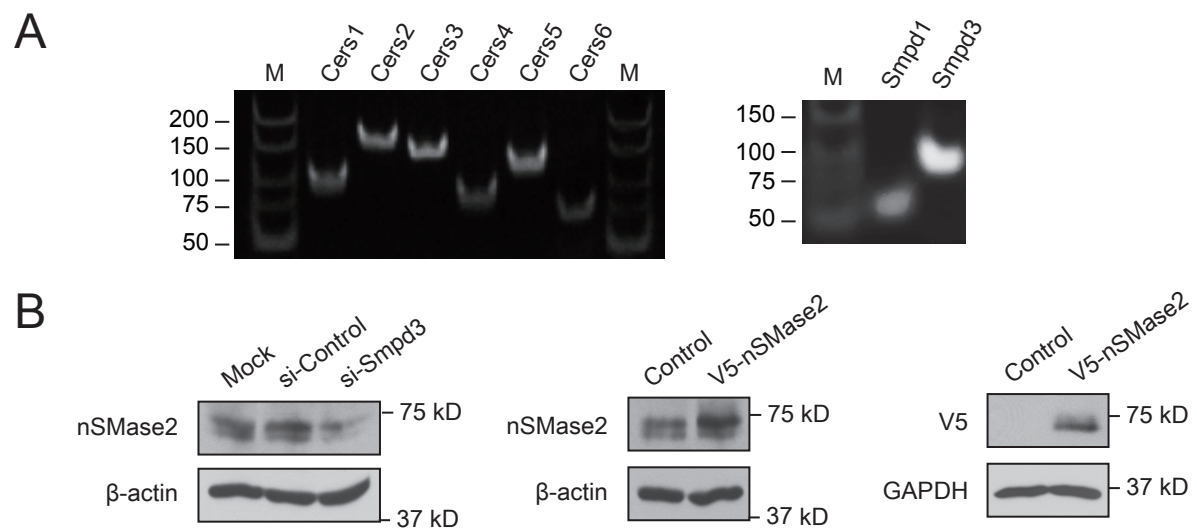

Figure S1

Supplement: Supplementary file 1 — Figure S1 [file 41419_2018_709_MOESM1_ESM.pdf]

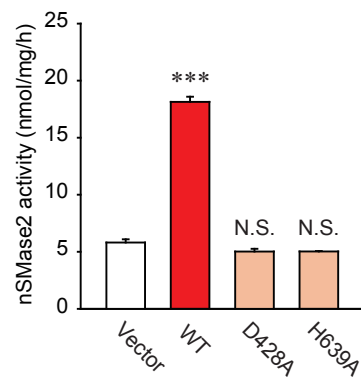

Figure S3

Supplement: Supplementary file 3 — Figure S3 [file 41419_2018_709_MOESM3_ESM.pdf]

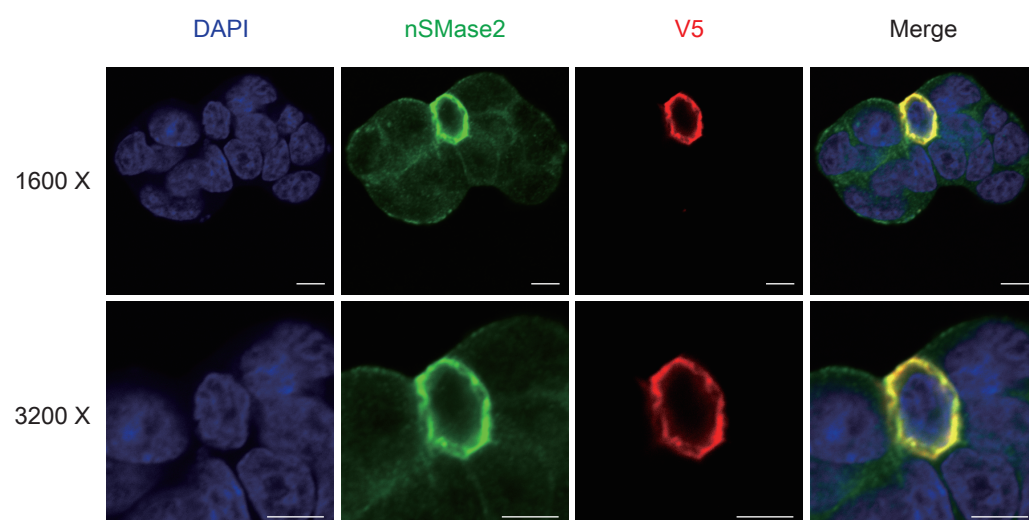

Figure S4

Supplement: Supplementary file 4 — Figure S4 [file 41419_2018_709_MOESM4_ESM.pdf]

A

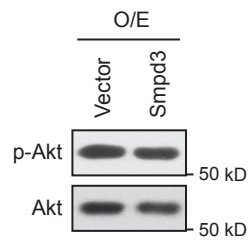

B

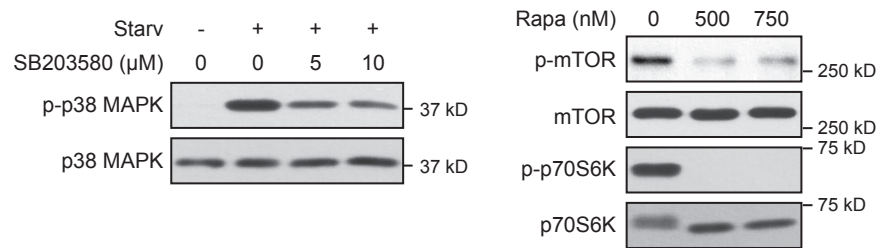

C

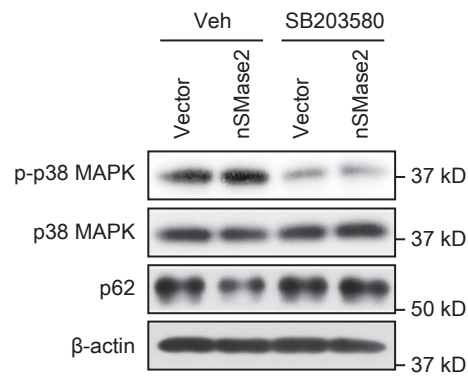

D

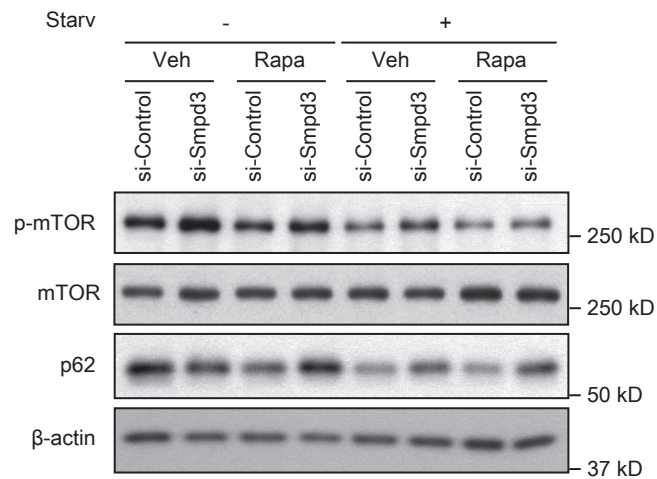

Figure S5

Supplement: Supplementary file 5 — Figure S5 [file 41419_2018_709_MOESM5_ESM.pdf]

A

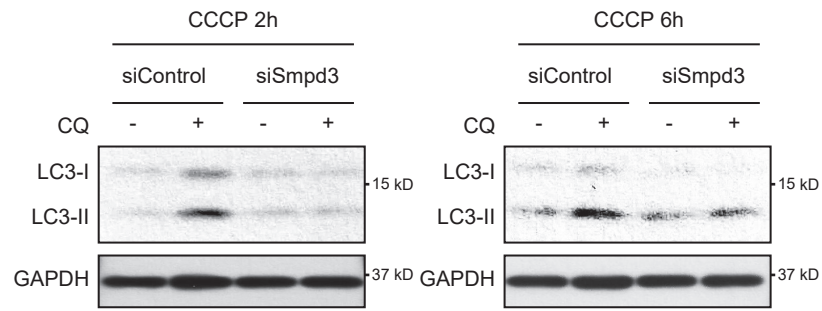

B

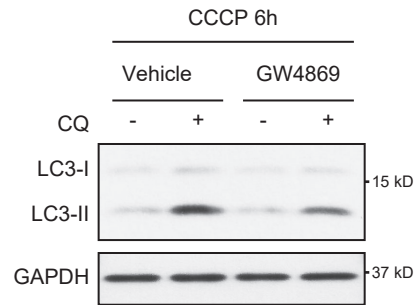

C

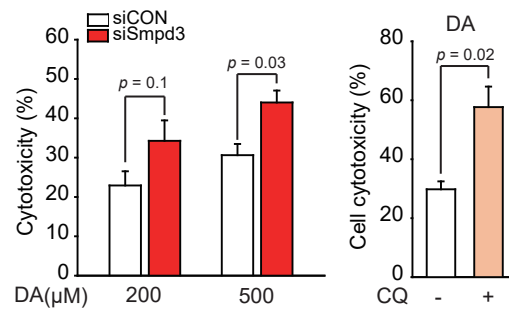

Figure S6

Supplement: Supplementary file 6 — Figure S6 [file 41419_2018_709_MOESM6_ESM.pdf]

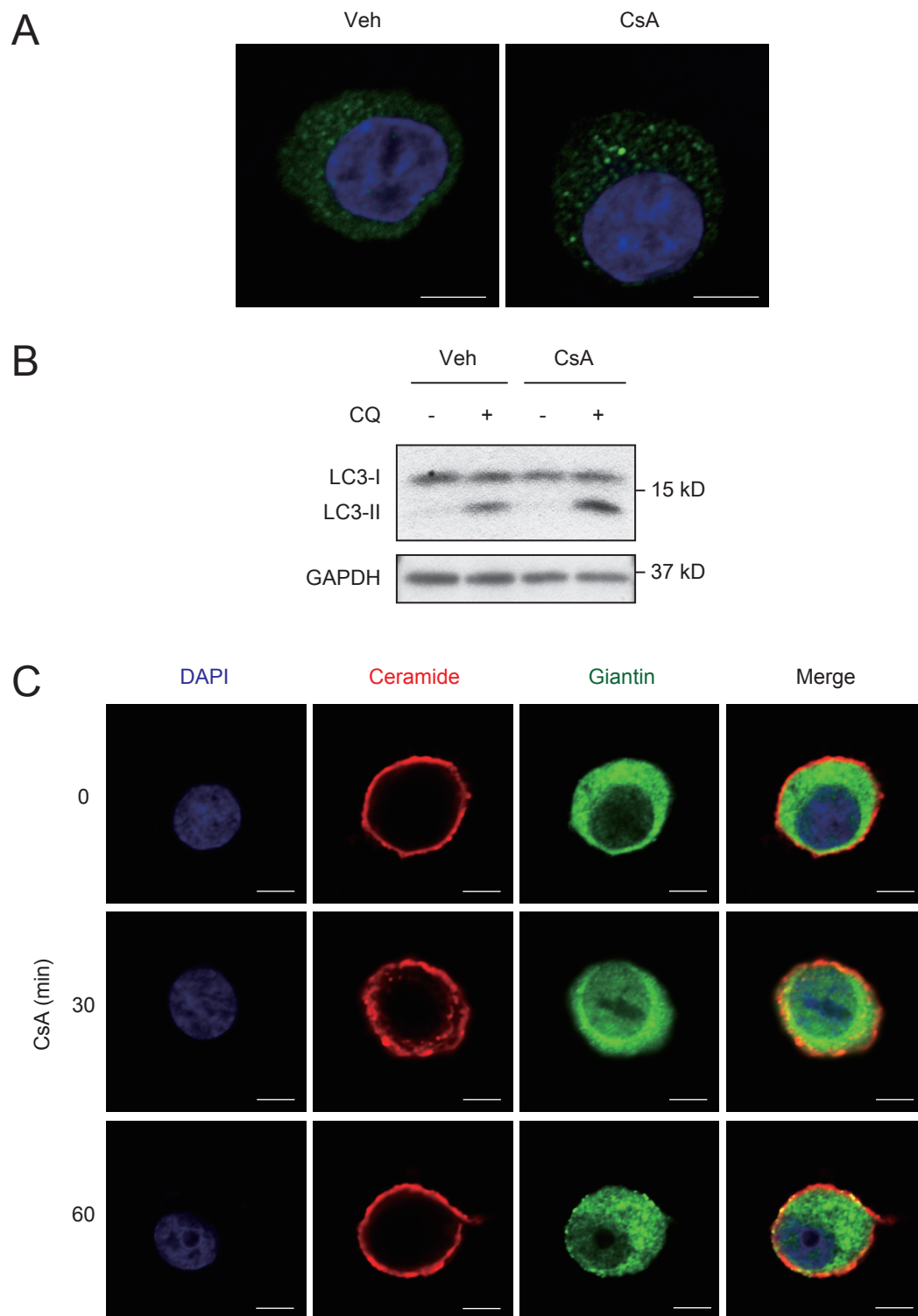

Figure S7

Supplement: Supplementary file 7 — Figure S7 [file 41419_2018_709_MOESM7_ESM.pdf]
