# Supplementary material for: Activation of neutral sphingomyelinase 2 by starvation induces cell-protective autophagy via an increase in Golgi-localized ceramide
Source: Cell Death Dis. 2018 Jun 4;9(6):670. doi: 10.1038/s41419-018-0709-4 (PMC5986760; doi:10.1038/s41419-018-0709-4)
Supplement: Supplementary file 2 — Figure S2 [file 41419_2018_709_MOESM2_ESM.pdf]

A

siRNA	Target sequence
#1	CAUCGACUACAUGCUGCACGCUGAA
#2	UGCUUCAGGACUGGCUGGCUGAUUU

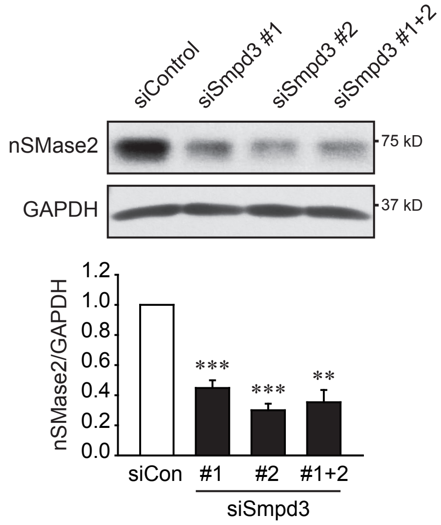

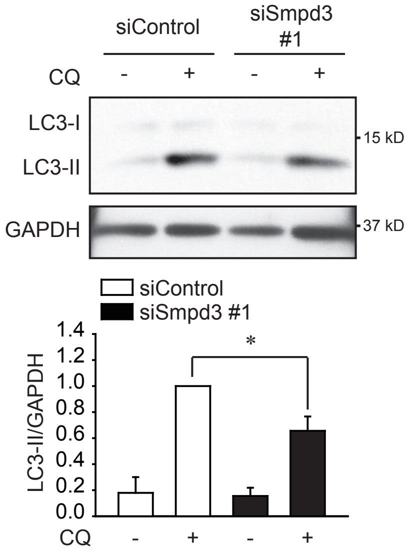

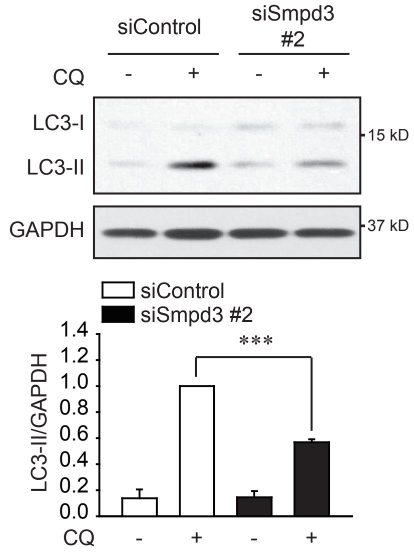

B

siRNA	Target sequence
#3	UCGCAAGGCUCAAUAAUGU
#4	GCGGAGAGCCAGGCAGUAA
#5	GGACAUAAACGGUCUCUAU
#6	GGGCAGAGAAUCCGCAAUG

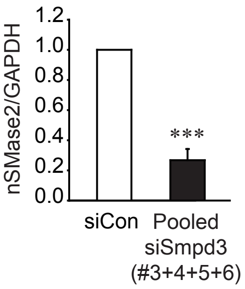

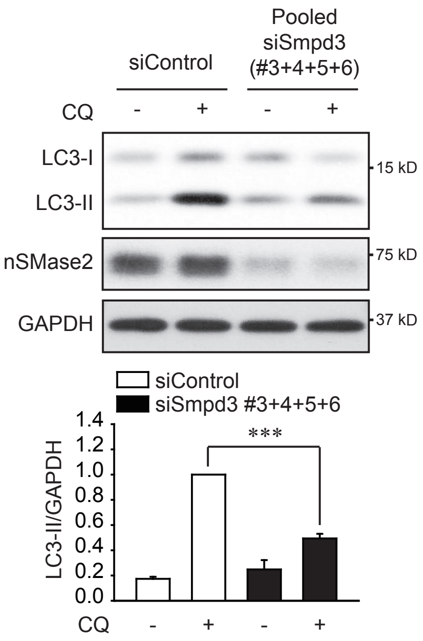

Figure S2
