# Supplementary material for: Activation of neutral sphingomyelinase 2 by starvation induces cell-protective autophagy via an increase in Golgi-localized ceramide
Source: Cell Death Dis. 2018 Jun 4;9(6):670. doi: 10.1038/s41419-018-0709-4 (PMC5986760; doi:10.1038/s41419-018-0709-4)
Supplement: Supplementary file 8 — Table S1 [file 41419_2018_709_MOESM8_ESM.pdf]

List of primers used in this study

Gene	Forward primer (5’-3’)	Reverse primer (5’-3’)	Amplicon size (bp)	Reference
CerS1	AGTGCCTGGAAGCTTCTGTT	GACCTCCAGCCGTAGAAGAC	116	Zuellig et al., 2014
CerS2	TTGAGGAAAGTTTGGGAAGG	AAACCAGGAGAAGCAGAGGA	188	Zuellig et al., 2014
CerS3	TGCCACACCTCTAGCCAATG	CCTGGCGCTCTGTCAAGTTA	158	Zuellig et al., 2014
CerS4	TCACTCTGCCCTTTGACATC	TGACGCTGTAGGAGAAGACG	96	Zuellig et al., 2014
CerS5	CACACAGCTGGCCTTCTACT	ACTCGCACCATGTTGTTGAT	141	Zuellig et al., 2014
CerS6	GGGATCTTAGCCTGGTTCTGG	GCCTCCTCCGTGTTCTTCAG	80	Zuellig et al., 2014
Smpd1	CAGTTCTTTGGCCACACTCA	CGGCTCAGAGTTTCCTCATC	65	Tong et al., 2010
Smpd3	TCTGCTGCCAATGTTGTCTC	CCGAGCAAGGAGTCTAGGTG	98	Tong et al., 2010
Hprt1	TGTTTGTGTCATCAGCGAAAGTG	ATTCAACTTGCCGCTGTCTTTTA	66	Seol et al., 2011

*Reference

ZUELLIG, R. A., HORNEMANN, T., OTHMAN, A., HEHL, A. B., BODE, H., GUNTERT, T., OGUNSHOLA, O. O., SAPONARA, E., GRABLIAUSKAITE, K., JANG, J. H., UNGETHUEM, U., WEI, Y., VON ECKARDSTEIN, A., GRAF, R. & SONDA, S. 2014. Deoxysphingolipids, novel biomarkers for type 2 diabetes, are cytotoxic for insulin-producing cells. Diabetes, 63, 1326-39.

TONG, M., LONGATO, L. & DE LA MONTE, S. M. 2010. Early limited nitrosamine exposures exacerbate high fat diet-mediated type 2 diabetes and neurodegeneration. BMC Endocr Disord, 10, 4.

SEOL, D., CHOE, H., ZHENG, H., JANG, K., RAMAKRISHNAN, P. S., LIM, T. H. & MARTIN, J. A. 2011. Selection of reference genes for normalization of quantitative real-time PCR in organ culture of the rat and rabbit intervertebral disc. BMC Res Notes, 4, 162.

Table S1
