# Supplementary material for: Activation of neutral sphingomyelinase 2 by starvation induces cell-protective autophagy via an increase in Golgi-localized ceramide
Source: Cell Death Dis. 2018 Jun 4;9(6):670. doi: 10.1038/s41419-018-0709-4 (PMC5986760; doi:10.1038/s41419-018-0709-4)
Supplement: Supplementary file 9 — Supplementary figure legends [file 41419_2018_709_MOESM9_ESM.docx]

**Figure S1.** (A) PC12 cell lines have a well-conserved sphingolipid metabolism. mRNA expression of *CerS* 1–6, *Smpd1* (aSMase), and *Smpd3* (nSMase2) was confirmed by RT-PCR. (B) Genetic knockdown of nSMase2 by transfection with rat *Smpd3* siRNA, and overexpression of nSMase2 by transfection with V5-tagged murine nSMase2 expression plasmid at 48 h after transfection. Protein expression of nSMase2, V5, β-actin, and GAPDH were analyzed by immunoblotting

**Figure S2.** Attenuated LC3 turnover by nSMase2 knockdown. (A) The target sequences of rat *Smpd3* siRNAs used in this study were described (Stealth siRNA; #1 and 2), and the levels of nSMase2 knockdown by a pool or each siRNA were determined. The decreases in starvation-induced LC3 turnover by individual siRNA were confirmed. (B) Verification of the suppression of nSMase2 knockdown on starvation-induced LC3 turnover using an additional pool of siRNAs (ON-TARGETplus SMART pool siRNA; #3, 4, 5 and 6). The data represent the mean ± SEM of three independent experiments. Significant differences between the indicated groups, *p < 0.05, **p < 0.01, and ***p < 0.001

**Figure S3.** Enzymatically inactive mutants were confirmed by nSMase2-specific activity assay using [^14^C]-sphingomyelin. The data of nSMase2 activity represent the mean ± SEM of three independent experiments. Significant differences between the indicated groups, ***p < 0.001; NS, not significant

**Figure S4.** The specificity of antibody binding to nSMase2 was confirmed by immunofluorescence assay in V5-tagged nSMase2-overexpressed cells. PC12 cells were transfected with plasmid-encoding V5-tagged nSMase2. At 48 h after transfection, assays using anti-nSMase2 and anti-V5 antibodies were performed. Yellow regions in the merged images represent binding to the same region by green fluorescent-labeled nSMase2 antibody and red fluorescent-labeled V5 antibody. Scale bar = 5 μm

**Figure S5.** (A) Unchanged level of Akt phosphorylation by overexpression of nSMase2. (B) The suppressed phosphorylation of mTOR and its substrate p70 S6K by rapamycin and the inhibition of starvation-induced p38 MAPK phosphorylation by SB203580 were verified. PC12 cells were pretreated with SB203580 or rapamycin at indicated concentrations for 1 h and then further incubated in the presence or absence of starvation for 2 h. (C) The effect of SB203580 on p62 degradation induced by nSMase2 overexpression. (D) The effect of rapamycin on si*Smpd3*-suppressed p62 degradation. Transfected cells were treated with SB203580 (10 μM), rapamycin (750 nM), or the vehicle for 1 h and then starved for 2 h. Phospho-Akt, Akt, phospho-p38 MAPK, p38 MAPK, phospho-mTOR, mTOR, phospho-p70S6K, p70S6K, p62, and β-actin levels were analyzed by immunoblotting.

**Figure S6.** (A) nSMase2 mediating autophagy induction by CCCP. PC12 cells transfected with siRNA were treated with 20 μM CCCP for indicated time. (B) Cells were treated with 5 μM GW4869 or vehicle in the presence of 20 μM CCCP for 6 h. CQ (50 μM) were untreated or treated for last 2 h. Autophagic flux was determined by LC3 turnover using immunoblotting. (C) The cytoprotective role of nSMase2 against high dose of DA exposure. The cells transfected with siRNA were treated with the indicated concentrations of DA for 24 h (left). To confirm the role of autophagy against DA exposure, cultured cells were treated with 500 μM DA for 24 h in the presence or absence of 50 μM CQ (right). Cytotoxicity was determined by the LDH assay.

**Figure S7.** (A) Representative images of LC3 puncta in cyclosporine A (CsA)- or vehicle-treated cells. PC12 cells were treated with 1 μM CsA or the vehicle for 2 h. LC3 puncta were detected by immunofluorescence assay using anti-LC3 antibody. (B) CsA-induced LC3 turnover. PC12 cells were treated with 1 μM CsA or the vehicle for 2 h in the presence or absence of 50 μM CQ. LC3 and GAPDH levels were analyzed by immunoblotting. (C) Increased co-localization of ceramides with the Golgi marker was triggered by CsA. Cells were treated with 1 μM CsA for the indicated times, and then co-stained with antibodies against ceramide and the Golgi marker giantin. Yellow regions in the merged images represent co-localization of green-stained Golgi and red-stained ceramide. Scale bar = 5 μm

**Table S1. List of PCR primers used in this study**
